# Gender-specific homophily on Instagram and implications on information spread

**DOI:** 10.1038/s41598-023-51117-w

**Published:** 2024-01-03

**Authors:** Yvonne-Anne Pignolet, Stefan Schmid, Arne Seelisch

**Affiliations:** 1 DFINITY, Zurich, Switzerland; 2 Weizenbaum Institute, Berlin, Germany; 3 TU Berlin, Berlin, Germany

**Keywords:** Computational science, Information technology, Scientific data

## Abstract

More and more social interactions happen online. On online social networks such as Instagram, millions of users share, like, and comment on photos and videos every day, interacting with other users world wide, at large scale and at a high rate. These networks do not only introduce new user experiences, but they also enable new insights into human behavior. Here, we use these new possibilities to study homophilic behavior—the tendency of individuals to bond with people similar to themselves. While homophilic behavior has been observed in many contexts, little is known about gender-specific differences and the extent of homophilic behavior of female and male users in online social networks. Based on a unique and extensive data set, covering over 800,000 (directed) Instagram interactions and a time span of three years, we shed light on differences between genders and uncover an intriguing asymmetry of homophily. In particular, we show that female users exhibit homophily to a larger extent than male users. The magnitude of this asymmetry depends on the type of interaction, as differences are more pronounced for ‘comment’-interactions than for ‘like’-interactions. Given these empirical observations, we further study the implications of such gender differences on the spread of information in social networks in a basic model. We find that on average, a piece of information that originates from a female group reaches significantly more female users than male users.

## Introduction

Online social networks and their explosive growth are arguably among the most disruptive trends of the new millennium so far^1–3^. On social networks such as Facebook, TikTok, or Instagram, billions of active users every month share, like, and comment on photos and other media content from users world wide^3,4^. The resulting scale and rate of human interactions is unprecedented.

For many people, these online social networks form an integral part of their daily routine and facilitate new user experiences^5^. Furthermore, the data produced in the process can potentially reveal new insights on human behavior. Indeed, a large body of research literature already provided many interesting results and observations of the relationships between online users^6–8^.

In this paper, we are interested in the homophilic behavior of users of the Instagram online social network. Homophily is an important and well-studied concept in sociology, revolving around the inclination of people to associate and form strong social connections with others who share one’s defining characteristics^9–19^.

While homophilic behavior has already been observed in many contexts, very little is known about the gender-specific differences and the extent of homophilic behavior of the female and male users in online social networks. Indeed, theoretical models for gender-specific glass ceiling effects often assume a symmetric homophily, where female users tend to associate with other female users to the same extent as male users associate with other male users^14^.

This paper revisits the question of gender-specific homophily, using a unique and large data set, covering over 800,000 interactions in the Instagram online social network, for a time span of three years. The data set is particularly interesting as interactions in Instagram are *directed* (i.e., asymmetric), from one users (female or male) to another user (female or male). Furthermore, the data set comprises two different types of interactions, *likes* and *comments*, allowing us to explore interaction-specific differences. Last but not least, the data set covers a significant time period, allowing us to study the evolution of homophilic behavior over several years.

Our paper uncovers significant differences between the genders and an asymmetry of homophily. In particular, we find that female users exhibit homophily to a larger extent than male users, and show that this asymmetry also depends on the type of interaction: the homophily of women is more accentuated for *comment* interactions. This is in contrast to empirically observed homophily among academics^17^, which is higher for male professors. We further observe an intriguing asymmetry between cross-gender interactions, and also answer questions related to which gender is more active, and which receives more likes/comments. For example, we observe that the origin of comments a male user receives is typically concentrated in a smaller set of female users, but is spread across a larger number of male users. Interestingly, also female users receive comments more often by the same female user while receiving more comments in total from different different male users. A similar picture also emerges for likes. In general, female users prefer to interact via likes more with female users while male users exhibit similarly interaction patterns with male or female users.

We further study the implications of gender differences on the spread of information in the social network, considering a basic model and using simulations on the graphs created by likes and comments. If a post from user A is being liked or commented on by user B, this indicates that user A has influenced the user B, by triggering this reaction. We observe that a piece of information which originates from a female group will reach significantly more female users than male users, which is due to the female users’ higher homophily and the overall larger number of female users. In contrast, a piece of information which originates from a male group will spread among male and female users alike. Male users spread information to a wider set of users on the graph describing comments interactions (a comment-edge is directed from the user who posted towards the other user who commented) while female users reach a larger spread on the graph formed by likes (a like-edge is directed from the user who posted towards the user who liked the post). A piece of information reaches significantly more male users relative to female users on the comment-graph compared to the like-graph. We also provide insights into the evolution of the Instagram network over time; in particular, we find that the density, i.e., the average number of interactions between users, diminishes with the increasing scale of the network.

Our observations hence reveal an interesting novel picture of homophily which complements existing literature. In particular, while homophily has been studied in many contexts already^9–14^, including its implications on glass ceiling effects^9,14^, we are not aware of any empirical work on the evolution of online social networks with different interaction patterns. Conceptually, our results on temporal development of homophilic tendencies and the asymmetry and concentration of *like* and *comment* interactions of average users are interesting. Furthermore, while the spread of information on networks has been subject to very intensive research^20–26^, we are not aware of any work on the implications of homophily on information propagation with the exception of^12,13^ both of which focus on the selection mechanisms for seed groups to meet certain goals. Finally, our work also complements the large body of literature on gender-based differences in other contexts^15,17,19,27^, including gaming^15^ and connections in a Spanish social network^19^ where a higher homophily among women has been observed before.

## Results

We observe several interesting differences in homophilic behavior between the genders. In the following, we report on the main results from our study of the Instagram online social network, revolving around *like* and *comment* interactions over a time period of several years. In particular, we first uncover interesting concentrations and biases in interactions, and then report on their implications on the spread of information in the network over time.

The data set was obtained by crawling the Instagram’s public profiles starting from the founder of Instagram, and covers 844,661 interactions (223,257 comments and 621,404 likes) over 160 weeks (until 10 May 2016)^10^. The data contains a list of interactions with source and target user IDs and a timestamp. The collected interactions reach 324,481 distinct target users.

### Which gender is more active?

**Figure 1:**
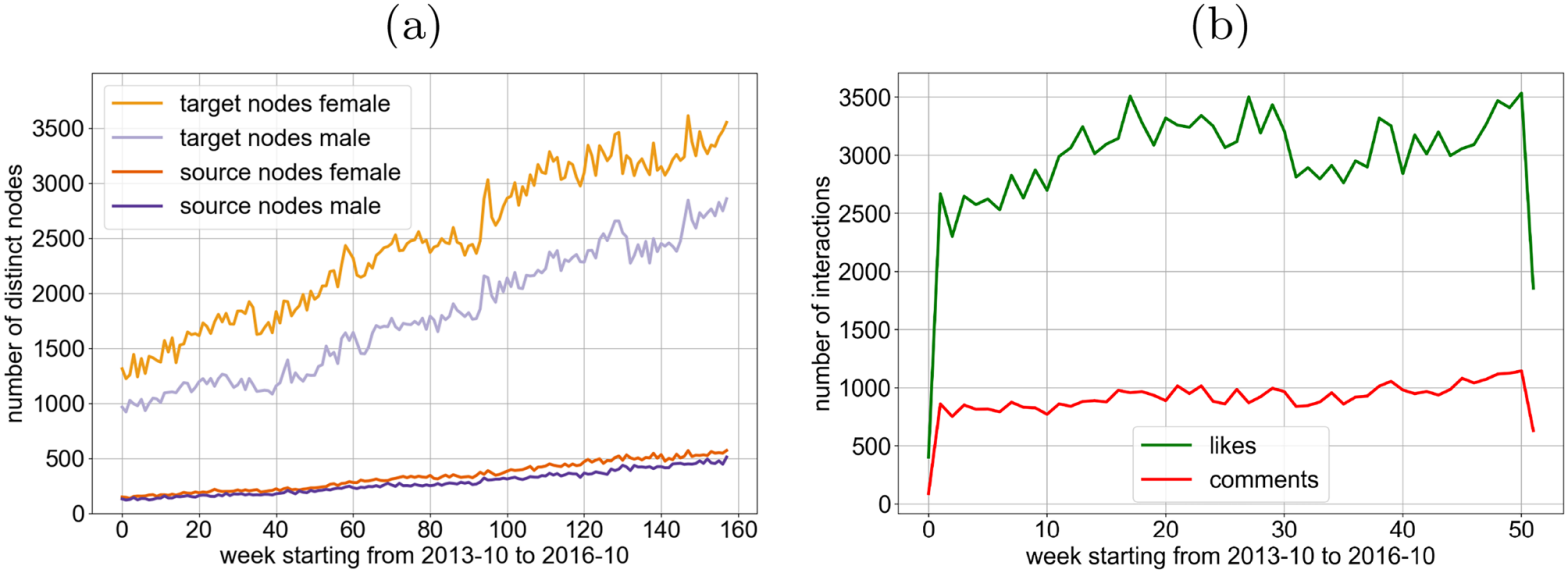
Growth of the Instagram network over the observed time, both in terms of (**a**) number of users and (**b**) interactions. Interactions (both likes and comments) are directed, from a source node who posted to a target node who reacted with a like or comment. Overall, female nodes appear more frequently both as source and as target. Furthermore, as expected, likes are more frequent than comment interactions.

We first provide some basic insights into the gender distribution in the data set. Table 2 lists the total number of distinct users of the respective gender, *female* and *male*, Fig. 1a illustrates the growth of data set over 160 weeks. Overall, there are more female users than male users; this is even more dominant for the target users of interactions compared to the source users who posted. While the number of users in the data set more than doubles over the observed period, the gender distribution remains stable.

**Figure 2:**
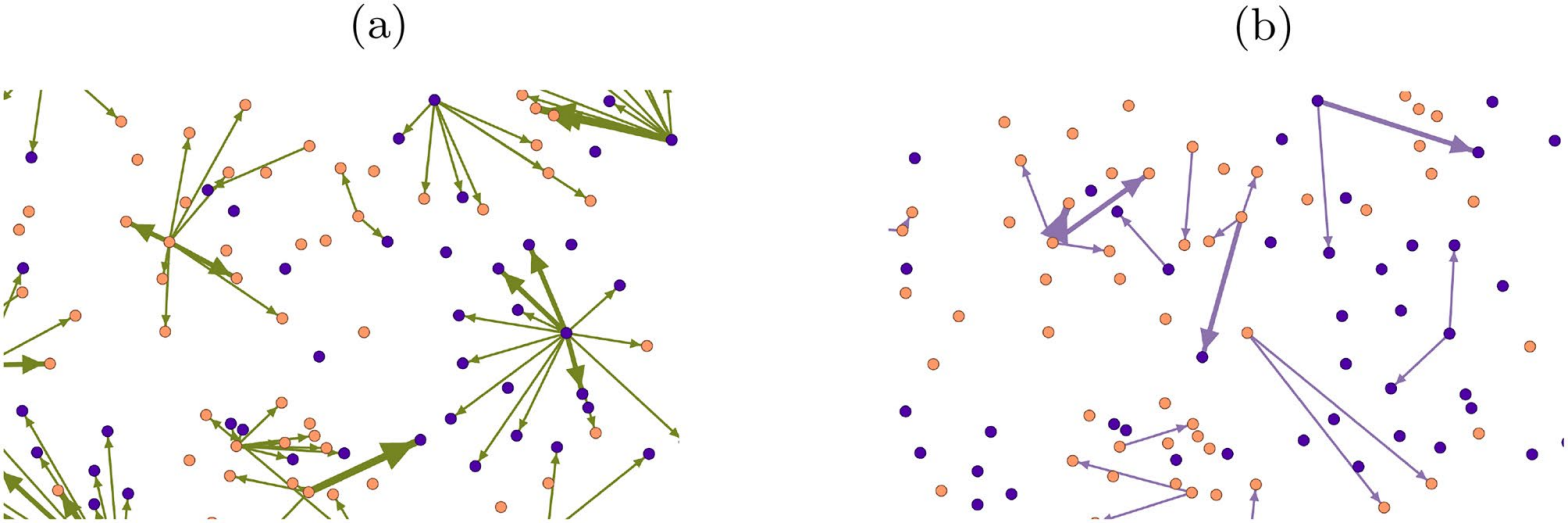
Visualization excerpt from snapshot of week 36 in 2015 with 6547 nodes and 5658 edges featuring 890 weakly connected components, zoomed in for a fraction of like (**a**) and comment (**b**) edges (directed towards the user who liked or commented). Female nodes (54.45%) are depicted in orange, male nodes (45.55%) in purple. The width of the edge illustrates the number of interactions. The graphs are very sparse, especially (**b**), with many connected components pre-dominantly featuring nodes of one gender.

As expected, there are more like interactions than comments (which generally require more effort to compose). The number of interactions grows at the same rate as the number of users. See Fig. 1b. An interaction is a *like* or *comment* for a post of another user, i.e., from a source node who posted to a target node who reacted. In our study we analyze graphs whose nodes are the active users and whose edges represent the interactions. A comment-edge is directed and points from the user who posted towards the other user who commented, and a like-edge is directed from the user who posted towards the other user who liked the post. We construct like and comment snapshot graphs as follows. We aggregate all like interactions of a given week into a snapshot graph where each node corresponds to one user and weighted directed links indicate the frequency of interactions between the two corresponding users in that week. Accordingly, we can define the in-degree resp. out-degree of a node (user) as the number of links pointing to it resp. away from it. See Fig. 2 for an excerpt from a snapshot for likes and comments, illustrating their sparseness and homophily.

**Figure 3:**
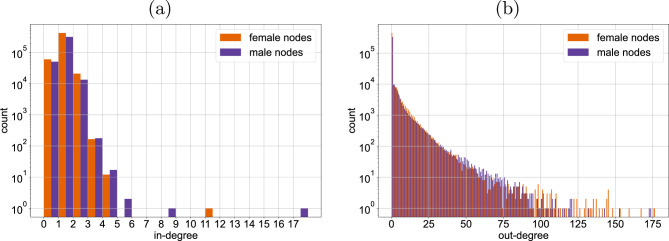
Interactions are skewed: users who send out a large number of interactions are mostly female. Moreover, there are slightly more male users with three or more incoming interactions from different users in the same week than female users. The in-degree (**a**) and out-degree (**b**) distributions over all snapshots are shown as barplots on a log-scale.

When studying the set of all snapshot graphs, in Fig. 3, we find that there are slightly more male users with three or more incoming interactions from different users in the same week than female users. While male users are the majority in the group with an out-degree of between forty and eighty, users that are the source of a larger number of interactions are mostly female.

### Homophily of like- and comment-interactions

**Figure 4:**
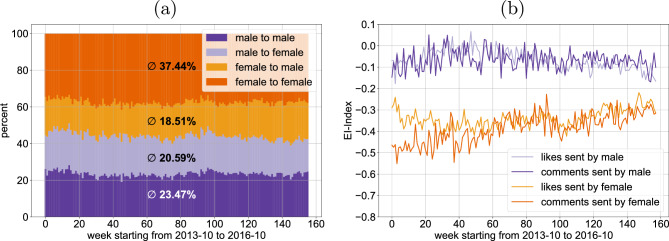
Women tend to interact more likely with other female users than with male users, both for likes (**a**) and comments (not depicted), based on weekly percentage of interactions with the same and opposite gender. This homophilic tendency is less pronounced among men. For both likes and comments, women interact with each other more than twice as often than with men, while men show only a light preference for interactions among themselves. EI homophily index per week (**b**), where 0 denotes no homophily and $-1$ denote pure homophily. Female users have a lower EI homophily index for the like- and the comment-graph alike, while male users have a higher tendency for equality. Female homophily is more pronounced for comments than for likes in the early weeks decreasing significantly in later weeks.

Figure 4a shows that the distribution of cross-gender interaction does not vary significantly in the observed period. The plot illustrates that women tend to interact more likely with other female users than with male users, both for likes and comments (figure omitted), while this homophilic tendency is less pronounced among men. In contrast to this finding, earlier work reports more cross-gender interactions from women in the context of scientific collaborations^14,28^. Since women are a minority in academia, and there are fewer men in this data set, the observed heterophily for the smaller group may be due to the relative size of the groups and not an intrinsic preference. Moreover, another explanation may be that selecting powerful collaborators (which are predominantly male in academia) helps career advancement, while Instagram interactions are often related to spare time activities and early stages of forming romantic relations.

In order to quantify homophily, we can consider the *EI homophily index* (representing a ratio of external to internal ties), also known as the Coleman homophily index^29^. This index is a relative measure since it does not take the underlying population into account and indicates the preference between external and internal groups. An external group for a user is the set of users of the opposite gender while an internal group is the set of users of the same gender. The EI homophily index *EI*(*g*) of a gender *g* in Fig. 4 was calculated with the following formula where $|E_{g\rightarrow \overline{g}}|$ is the number of edges that start at a user of gender *g* and end at a user of the opposite gender $\overline{g}$.

$$\begin{aligned} EI(g) = \frac{|E_{g\rightarrow \overline{g}}|- |E_{g\rightarrow g}|}{|E_{g\rightarrow \overline{g}}|+ |E_{g\rightarrow g}|}. \end{aligned}$$

An EI homophily index of $-1$ indicates pure homophily since it means that all edges are only between users of the same gender. On the other hand an index value of $+1$ indicates pure heterophily since in that case all edges are between users of different genders.

In Fig. 4b we can see that female users have a lower EI homophily index for the like- and the comment-graph alike. The first 80 weeks exhibit more volatility since the data set provides less than 1500 comments and less than 4000 likes per week in that time-frame.

It is clearly visible that male users have a higher tendency for equality than female users. Looking at the EI homophily index of the comment-graph this tendency for homophily becomes even more pronounced between week 80 and week 200. This shows again that female users’ posts trigger more comments from other female than from male users, while the threshold for likes is smaller and therefore the EI homophily index is slightly higher in the like-graph.

### How are interactions concentrated?

**Figure 5:**
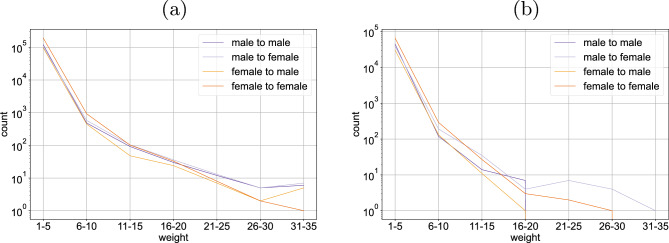
Male users tend to interact via comments more often with the same female user while their comment interactions are more spread among different male users. For female users it is the other way around. Generally, male users have higher weights for comments between a male and a female user than between two male users. For likes the differences are less pronounced. (**a**) Weights distribution for the like-graph genderwise over all snapshots (20 outlier edges with weights larger than 35 omitted). (**b**) Weights distribution for the comment-graph genderwise over all snapshots.

It is also interesting to study how users distribute their interactions across other users. In our weekly snapshot graphs, we represent the frequency of an interaction in that week by the edge weight. Figure 5 shows that the maximum number of the interactions between a pair of users can be fairly high, although the average itself is low.

We observe that there are always more edges of a weight for comments between two female users. Interestingly we can also see that male users have higher weights for comments between a male and a female user than between two male users. So it appears that male users tend to elicit comments more often from the same female user while they receive comment more often to different male users. For female users in comparison it is the other way around. They tend to get comments more often from the same female user. The like-graph (Fig. 5a) gives a similar picture as the comment-graph (Fig. 5b). There are more likes triggered by a male users from the same female user than by a male from the same male user, and more likes triggered by a female from the same female user than by a female from the same male user. Female users prefer to interact also via comments more with female than with male users while male users tend to interact almost similarly with male or female users.

### How does homophily impact the spread of information?

One of the key aspects studied in many social networks concerns the spread of information^20–26^, however, very little is known about the implications of homophily on information propagation, especially from an empirical point of view. Our data set allows to provide insights into this aspect. Concretely, in order to study the impact of homophily on the propagation of information, we implemented a simulator which we can feed with our empirical data (the Instagram interactions) discussed above. As a first step, we consider a very simple model. The simulator selects a starting group that knows a piece of information and then disseminates it to other users through the edges of the weekly interaction graph snapshots (in our case from Instagram). The dissemination process follows the well-known SIR (susceptible-infected-removed) model also studied in epidemics^30^. We emphasize that there exists a large body of literature and more realistic models for information spreading processes (e.g.,^31–33^), especially for capturing social complexity behind diffusion, whose study however we leave for future research.

To compare differences between female and male users when propagating information. we use different starting distributions for the starting group. In particular, we consider a start group of 100 users without any female users, one with a 50% distribution and one with only female users, chosen uniformly at random among all users of the target gender. We performed simulations on the unweighted like-graph and comment-graph.

**Figure 6:**
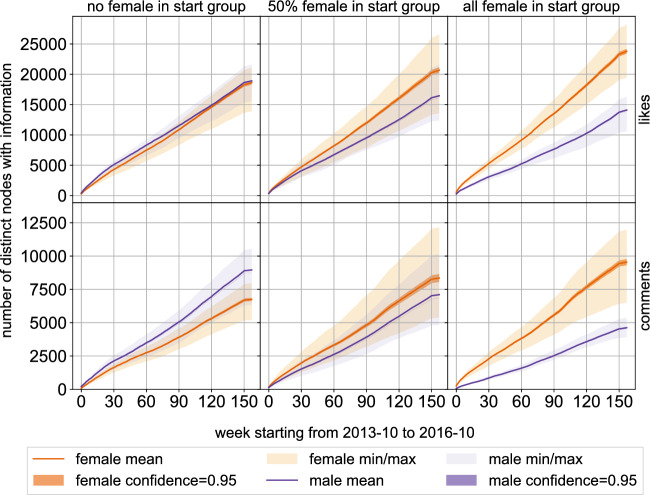
The figure shows dissemination on like- and comment-graph with an all-male, a 50% female, and an all-female start group. Y-axis shows the number of distinct users with information per week. Information generally reaches more users with all-female start groups, both with like interactions and with comment interactions.

Figure 6 depict the number of distinct users which have obtained the information, per week after 100 runs each on the comment-graph and like-graphs respectively for (a) an all-male, (b) a 50–50 distributed, and (c) an all-female start group. For an all-male start group, we can see that on average more male than female users got the information, and the reach is lower than for the other starting groups. If we start with an all-female group, more female than male users know the information at all times and the difference between the average number of users reached is visibly larger than with an all-male start group. Since female users tend to higher homophily than male users in this data set and the total number of female users is higher, the number of female users with the information is higher than the number of male users.

Qualitatively the results on the like-graph look understandably similar to the results on the comment-graph, and homophily effects can be seen. As expected from the higher number of likes, the information reaches more users on the like graph (almost twice as many) compared to the comment graph. Another difference can be seen when comparing the plots with all-male starting groups, where the gender distribution on the like graph is almost 50%, although their homophily index for likes and comments is the same. The figure also shows that despite starting with an all-male group, the number of female users with the information grows to almost the same number in the end. This is explained by female users favouring likes as interaction and giving the majority of likes overall.

**Table 1:** Average (95% confidence) depth and breadth for start groups with 0, 50% and 100% female users. The depth for likes is significantly higher than for comments independent of the groups. Similarly, the breadth difference for likes and comments is very pronounced. Breadth values at the start exhibit homophily.

Start group	No female	50% female	All female
Depth likes	4.38 (0.13)	4.15 (0.07)	4.22 (0.10)
Depth comments	2.93 (0.05)	2.99 (0.01)	2.96 (0.04)
Start breadth likes	94.57 (0.38)	91.83 (0.31)	97.28 (0.18)
End breadth likes	70.38 (1.27)	63.67 (0.66)	66.93 (0.88)
Start breadth comments	64.81 (0.76)	63.74 (0.46)	64.99 (0.48)
End breadth comments	38.98 (1.01)	44.81 (0.60)	32.08 (0.57)

There are several additional interesting metrics to characterize the diffusion effect of information^34^, besides the number of nodes, in particular the *depth* and *breadth* of the dissemination process. We study these two metrics next. More precisely, we compare the maximum number of hops from the starting groups the information has travelled as well as the maximum number of users involved in the dissemination at any time, i.e., how many neighbors of newly informed users had the information in earlier rounds in Table 1. With an average value of more than four, the dissemination on the like graph is much deeper than on the comment graph with an average value of less than 3. Analogously, the breadth at the beginning and at the end of the dissemination on the like graph is with over 90 in the first round and over 60 in the end significantly higher than on the comment graph, which start with a bit more than 60 and ends above 30 nodes. In the all-female start group more nodes are involved in the beginning for both comments and likes compared to the all male and mixed groups, towards the end, the situation changes. This indicates that due to the homophily, the information spreads more quickly in the former groups, but also that the dissemination activity slows down faster.

An interesting observation is the fact that the ratio of users with the information to all users in the network is declining over time. This means that the number of users with a piece of information is growing slower than the total number of users in the network. So there might be an upper limit to the spread of information in this kind of network.

## Discussion

This paper uncovered a significant asymmetry of gender-specific homophily in Instagram, one of the largest online social networks. In particular, based on an extensive empirical study covering hundred thousands of user interactions, we find that female users exhibit homophily to a larger extent than male users, especially for *comment* interactions, but also for *likes* interactions. We further observe that the comments of a male user are typically concentrated on a smaller number of female users, but are spread across a larger number of male users. Also female users comment more often on items by the same female user while commenting more on different male users. A similar picture also emerges for likes. Furthermore, our work sheds light on the evolution of the Instagram network over time, and we show that the density, i.e., the average number of interactions between users, diminishes with the increasing scale of the network.

Our paper hence complements existing literature on homophily^9–14^. In particular, in contrast to Instagram, homophily among Polish academics is stronger for male professors^17^, and in this regard the Instagram network resembles more networks known from gaming applications^15^.

We also provided insights into the implications of gender differences and asymmetry of homophily on the spread of information in the social network. We have shown that a piece of information which originates from a female group will reach significantly more female users than male users. In contrast, a piece of information which originates from a male group will spread among male and female users alike when using the like-graph. Male users spread information to a wider set of users on the comment-graph while female users reach a larger spread on the graph formed by likes. A piece of information reaches significantly more male users relative to female users on the comment-graph compared to the like-graph.

The spread of information on networks has been subject to very intensive research^20–26^, and also homophily aspects have been studied in the literature before, for instance, related to the role of homophily on misinformation and polarization^35,36^, or the spread of political information on social media^37,38^, e.g., on the subject of immigration^39^, however, we are not aware of any similar work on the implications of homophily on information propagation on Instagram.

We believe that our work can hence inform policy makers about the influence of homophily on important properties of online social networks such as Instagram, including how and how quickly information can spread. Such information may also include rumors or even software viruses, and hence be a factor for explaining phenomena like filter bubbles which have recently received much attention, e.g., during the pandemic. Our insights may also have implications on marketing strategies, aiming to reach a maximal number of users efficiently.

We hence understand our work as a first step and believe that it opens several interesting directions for future research. In particular, it would also be interesting to investigate whether similar trends also exist in other social networks, such as TikTok (unfortunately, very limited data is available to researchers for such social networks). Furthermore, we have focused on a very simple model for information propagation, and it would be interesting to extend our study to other models. In particular, while compartmental models such as the SIR model are useful in epidemiology, it will be interesting to study more complex models specific to the information spread^31–33^, allowing to take into account social aspects.

## Methods

We first report on the data set and then elaborate on our simulation methodology. We also discuss some limitation of our methodology. Our methodology is based on publicly available user profiles which have been anonymized, an on model simulations on top of the resulting graphs. We confirm that our work does not raise any ethical issues.

### Data sets and empirical methodology

Our evaluation is based on a large data set collected from the Instagram online social network^10^ (Table 2). The data set was obtained by crawling the Instagram’s public profiles starting from the founder of Instagram.

Stoica et al. used the Instagram API to gather profile information by recursively crawling the lists of followers. For each profile, the username was stored and the meta-data for each photo processed to include timestamps a random subset of up to 5 likes and comments with their authors.

**Table 2:** Total distinct users per gender in data set from Instagram used for our study.

Gender	Source		Target	
	Number	Share (%)	Number	Share (%)
Female	3690	53	178,749	55
Male	3271	47	145,732	45

Overall, the data set contains 999,998 interactions in which the gender is present for both users, and spans from the mid 2010 (when Instagram was launched) to Summer 2016, 305 weeks in total. This is an interesting time period in which the network grew significantly. The measurements had to be discontinued in 2016 as crawling the online social network was made increasingly difficult to researchers. In our analysis we focus on the last 160 weeks (until 10 May 2016), since the first 3 years are noisy due to the high growth rate of the network. In this time frame, there are 844,661 interactions, more precisely 223,257 comments and 621,404 likes were collected.

The data contains a list of interactions with source and target user IDs, and a timestamp for each row. The source denotes the poster of a photo, the target is the person who liked/commented. For each user ID their gender was guessed based on the self-reported name and the provided social security data, which is consistent with the methodology used before in the literature^10^. There are 6961 distinct source users and 324,481 distinct target users. The ratio is around 1:47 which shows that there are a lot more receivers than senders, which is also due to the recursive method with which the data set was obtained. Restricted to users with more than one interaction shows that there are 6498 distinct source users and 114,969 distinct target users.

We note that the data set collected by Stoica et al.^10^ does not contain all interactions between users per photo but a randomly sampled subset of up to 5 interactions for each processed photo.

Also, while there is a spectrum of gender identities in reality, the data set uses a binary assignment to a subset of the users, which may disagree with the user’s identity conception. The gender was derived by Stoica et al.^10^ leveraging profile first name and social security name data between 1940 and 2007. Reported names with less than 50 occurrences for either men or women and names with less than 95% of single gender were excluded. This created 32,676 unique unambiguous first names, which were used to label 92,935 Instagram users (38% of the all users), for which the authors report a 97% accuracy based on manually checking a small test sample.

Our data set also comes with some additional limitations. Our results are not only influenced by the sampling method but also by the choice of the starting node. While we observe a long period, with a significant growth, this initial starting node may still play a major role. Also, the interaction density is relatively low, even for a period of time where Instagram’s size was not comparable to its current size. Also, the imbalance between number of senders and recipients has an effect on the generality of the results, and it will be interesting to study alternative time periods and data sets.

### Simulation methodology

We use a dynamic graph model to analyse the temporal evolution of the Instagram social network. For each week in the data set we construct a snapshot graph with active users as nodes and a directed edge between them if an interaction took place between them. We apply the the well-known SIR (susceptible-infected-removed) model^30^ to simulate the spread of information on the evolving network. A piece of information can spread from node *a* to an adjacent node *b*—a neighbor of *a*—if *a* knows the information and *b* decides to believe the information. After a successful transfer both nodes involved know the information and can spread it further on the graph. Depending on the precise model variation chosen, the probability for a node to adopt and propagate a node’s information may be probabilistic and depend on their edge weight, the number of times the information has been received or other factors. In our simulations we proceed in rounds and a piece of information is always successfully spread to all neighbors in a snapshot. For the next round, the next snapshot is used.

The information dissemination simulations were executed on a virtual server with eight cores, allowing for one simulation run of the Instagram data set (roughly one million rows) within 26 minutes. We performed 100 runs per parameter combination to provide a large enough sample and analyse distributions. The 95 percent confidence interval was typically very narrow around the mean, while the minimum and maximum values differed significantly.

### Consent to participate

The data used in this article was acquired via a public API by Stoica et al.^10^.

## Data Availability

The datasets analysed during the current study are available in the Zenodo repository (https://zenodo.org/record/8228788).

## Code availability

The code developed for the current study is available in the Zenodo repository (https://zenodo.org/record/8228788).
